# Insights from the molecular docking analysis of gambogic acid with the Chikungunya spike glycoprotein E2

**DOI:** 10.6026/97320630019525

**Published:** 2023-05-31

**Authors:** Sadia Qamar, Amna Syeda, M Amjad Beg, M Irfan Qureshi

**Affiliations:** 1 Proteomics and Bioinformatics Lab, Department of Biotechnology, Jamia Millia Islamia, New Delhi 110025; 2 Centre for Interdisciplinary Research, Jamia Millia Islamia, New Delhi 110025

**Keywords:** Chikungunya, spike protein E2, binding energy, gambogic acid, docking, healthcare

## Abstract

Chikungunya fever is a mosquito-borne disease caused by the chikungunya virus (CHIKV) and has drawn substantial attention in recent years. So far, no effective treatment is available in the form of drugs or vaccines. In this study, we aimed to screen some drugs against the pathogenic Chikungunya virus through a molecular docking approach. As a fact, the spike E2 protein plays an important role in viral attachment to the human host cell, binding to a cell receptor MXRA8. The molecules screened for in-silico interaction against MXRA8 were selected with top hit based on binding affinity. The existing intermolecular bonds were investigated further in the active site of the protein that interacts with the top-hit ligands. Gambogic acid (guttic acid) was depicted as the furthermost potential inhibitor when compared to the others it had the lowest binding affinity (-10.9 kcal/mol). Gambogic acid, as a potential antiviral agent against the spike E2 protein, could be a promising candidate.

## Background:

The emergence and re-emergence of the Chikungunya disease caused by the Chikungunya virus (CHIKV) are public health concerns worldwide. Chikungunya virus is an alphavirus that is transmitted by mosquitoes of the Aedes species and belongs to the family of Togaviridae [1]. The word Chikungunya arises from the word Makonde which means "bends up" i.e. posture due to severe pain in joints. The Chikungunya virus (CHIKV) was first identified in Tanzania in 1952 [2] and later developed as an outbreak on the French Island of Reunion in 2005 [1]. CHIKV has spread to more than 40 countries so far including Africa, Asia, and Europe over the past decade, causing more than a million infections in the Americas alone since 2014 [3]. In recent years, the worldwide incidence of chikungunya viral fever has dramatically increased. Rashes, myalgia, high fever, and, usually extreme arthritis are among the signs of the disorder [4]. The CHIKV is enveloped, spherical, positive single-stranded RNA virus which is about 60-70 nm in diameter [5,6]. CHIKV is about 11.7 kb and has capped at 5' and poly-A tail at 3'. The genome of CHIKV contains two open reading frames (ORFs) that encode for two polyproteins (structural polyprotein and non-structural polyprotein), which at 3' cleaved into five structural proteins (Capsid, E3, 6K, E2 and E1 glycoproteins) and four non-structural proteins at 5' (nsP1-4) by cellular and viral protease [7]. E2 is mainly responsible for cellular receptor interactions and E1 promotes endosome virus fusion. There are no known therapies for CHIKV infections at present and no approved vaccine is available for human use for any alphavirus. Gambogic acid is a natural compound derived from the resin of the Garcinia hanburyi tree, also known as gamboge. It has been traditionally used in Asian medicine for its potential therapeutic properties. However, it's important to note that gambogic acid can be toxic at high doses [8]. Therefore, it is of interest to document the molecular docking analysis of gambogic acid with the Chikungunya spike glycoprotein E2.

## Methods:

## Retrieval of the protein sequence:

All analyzed protein sequences were retrieved from the UniProt database (https://www.uniprot.org/). One of the CHIKV spike glycoprotein E2 is a structural polyprotein of the Chikungunya virus. By binding to the cell receptor MXRA8, E2 mediates attachment of virus to the target host cell [9]. Protein sequences were obtained in FASTA format and used for further studies.

## Homology modeling and validation of structure:

The Swiss Model (https://swissmodel.expasy.org/) is online available for predicting the homology modelling of the protein. It is used to generate 3D model of Chikungunya virus Spike glycoprotein E2. To estimate the homology modelling using the FASTA format of the amino acid sequence while this web-cut-off server's calculation is different, implying that the properties from the target-template alignment and the template structure energy analysis and QSQE and GMQE, as a qualitative model energy analysis method and quality estimation method that incorporates properties of described the major geometrical aspects of protein structures [10]. The model evaluation was carried out by the PROCHECK server, and the Ramachandran plot depicted the overall geometry of the model valuation, which specifies whether the measured score would interpret the modeled protein structure as being in the preferred, permitted, or prohibited area. 90 percent scores will be considered a high-quality model in the most favored area [11].

## Molecular docking (SBVS method):

As an outcome, the ligands are categorized based on their binding free energy (kcal/mol) for the target, with the most promising compounds being listed top of the (Table 1). In this method SBVS, compounds are taken from a large database subset which is categorized based on their binding free energy for the receptor site [12]. For molecular docking through SBVS prepare both files as PDBQT. To prepare the pdbqt file all water atoms were deleted, adding hydrogen bond polar only, and adding charges of both receptor and ligand. Using our published protocol, we converted the receptor protein (target) from a .pdb file to a pdbqt file [13]. The ZINC database subset Herbal Ingredient Target (HIT) was used to get the compounds library. PyRx method was used to minimize the energy of these small molecules (natural compounds) and transform this SDF format into PDBQT. For this simulated screening of spike E2 protein and HIT compounds using InstaDock v1.0. Using the hybrid scoring feature in InstaDock software, the binding free energies between the ligand and the protein were determined. The protein was held rigid in this analysis, while the ligands were completely flexible. The active binding pocket of the spike E2 protein structure was predicted by the CASTp server, ensuring that docking was efficacious. The best-fitting conformation relating to the binding affinity of the ligand-receptor complex was recognized thus keeping the receptor rigid and the ligands flexible. The top ten hits binding free energies compounds against Spike E2 protein were selected [14]. Using the rule of five (Lipinski's rule), the drug-ability analysis was carried out using the online cheminformatics program SwissADME to see whether the compound met the conditions for a drug candidate and the prediction of ADMET parameter analysis of the chosen compounds was also determined using the admetSAR server. From the database of docked ligands, the ligand with the lowest binding free energies was chosen which investigate the molecular field characteristics and bound conformation coordinate of the Spike E2 protein complex using Discovery Studio 2019. This output visualization analysis in two-dimensional structures explored the interacted residues [15].

## Results:

Spike glycoprotein E2 was selected for this analysis because it is involved in viral attachment to target host cells which play a prominent role in providing entry to Chikungunya virus. The sequence was obtained in FASTA format from UNIPROT whose protein ID is Q8JUX5 used for further study.

## Homology modelling and validation of structure:

**Figure 1:**
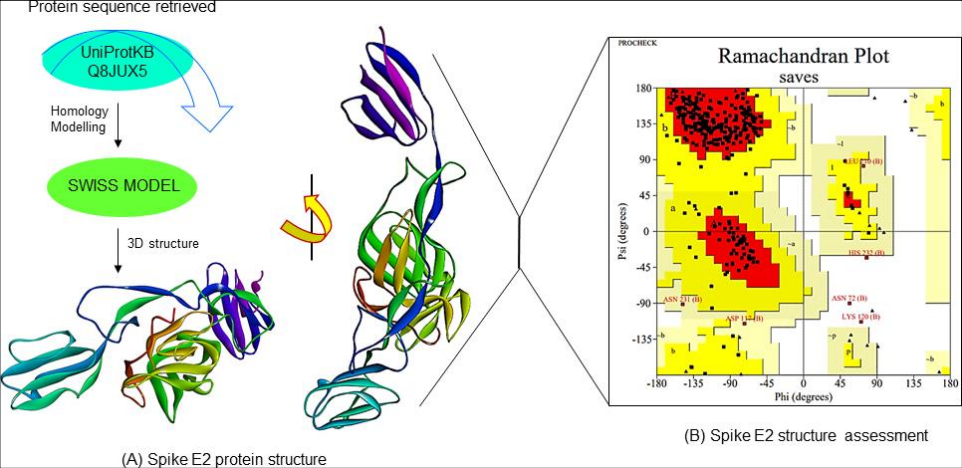
Homology modelling and validation of structure: (A) the spike E2 protein modelled by homology modelling using SwissModel. (B) Ramachandran plot studied favoured and allowed/disallowed regions using PROCHECK server

The sequence obtained from UniProt with the accession number Q8JUX5 was used in the current studies for molecular modeling of spike E2 protein. The Q8JUX5 predicted model was based on template-based homology modelling. After the acquiescing on the best amino acid sequence template is 2XFC which description has E2 enveloped glycoprotein and the sequence identity is 96.69% where the template coverage is ~80%, the predicted form is monomer oligo state with no ligand bound to it, the GMQE value is 0.68 and QMEAN value is -0.19 which means the modeled protein is showing satisfactory results and the 3D structure was shown in Figure 1. The PROCHECK server was used to validate the model, and a Ramachandran plot analysis of the projected model revealed that the modeled structure of spike E2 protein had ~90% residues in the most favored field, which means the modelled structure is likely to be good else values were shown in Figure 1.

## Molecular docking (SBVS method):

**Figure 2:**
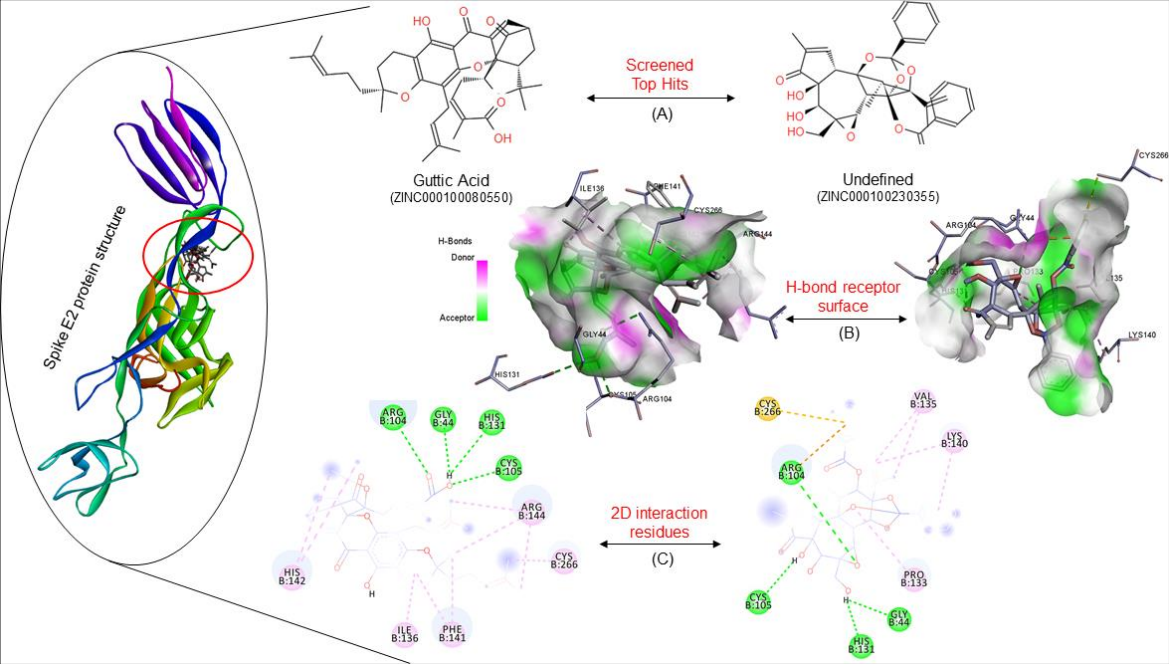
Structure of Spike E2 protein with top hits ZINC000100080550 and ZINC000100230355. (A) Chemical structure of the top hit compounds. (B) The complex structure shows the presence of hydrogen bonds interacting residues. (C) Finally, the 2D interaction analysis shows the complex file interacted sites with different colours showing the bond are Purple_Pi-Alkyl, and Green_Hydrogen bond.

The molecular docking-based SBVS method using InstaDock v1.0 software it includes the fundamental orientations between the receptor and the drug. Furthermore, the Spike glycoprotein E2 was used for molecular docking to explain how these proteins interact with the drug compound and then studied the drug scan as shown in Figure 2. and the docking score lower binding free energy kcal/mol was strong evidence to indicate that their complex binding is steady. The top 10 dockings hit obtained from the Virtual screening of Virus E2 protein with (Herbal ingredient target) database was shown in Table 1. The top ten hits with binding free energies ranging from (-10.9 to -9.9 kcal/mol) were chosen for further studies. ZINC000100080550 has a higher binding score than Spike E2 protein and the HIT database in this screening.

## Molecular field analysis:

The interaction analysis of the three-dimensional complex structural study of the 3D crystal structure of spike glycoprotein E2 with selected compounds (ZINC000100080550 and ZINC000100230355) was carried out in the active site binding interaction studies. Docking analyses of selected compounds were conducted to determine the binding association of compounds with active sites of the protein which revealed that these ZINC000100080550 and ZINC000100230355 compounds occupy active pockets of the protein were shown in Figure 2. Interaction studies showing the ZINC000100080550 interacted with binding residues were GLY44, ARG104, CYS105, HIS131, ILE136, PHE141, HIS142, ARG144, CYS266 and ZINC000100230355 interacted with similar amino acids except for HIS142 and ARG144 but it has some other attracted alkyl hydrophobic interactions which are PRO133, VAL135 and LYS140. ZINC000100080550 is known as a Gambogic acid or Guttic acid; it acts as a potential inhibitor of the best pharmacokinetics, drug-like effects, active site engrossment, and a variety of pharmacological properties.

## Binding energy determination and drug-ability assessment:

The drug-likeness properties of selected compounds were assessed using the SwissADME server which reveals the significant characteristic of drug action in a curative manner as conical smiles in its input system. In a drug repurposing, ADMET will be able to design and refine lead compounds and it was done by the admetSAR server. Finally, the selected compound ZINC000100080550 accomplishes four in five ADMET principles except for Excretion output interpretability because it acts as a promising target against Spike glycoprotein E2 and these ADMET properties was shown in Table 1.

## Discussion:

Since there were no experimental structures reported for Spike E2 protein yet. SWISS-MODEL was used to predict the homology model for the three-dimensional structures of the protein. The 3D structure allows one to consider the binding specificities of the ligand, which play a crucial role in studying protein-ligand interactions. The most accurate statistical approach for generating reliable structural models was homology modelling. After the homology modelling the stereochemical quality of the predicted model was assessed using Ramachandran plot calculations computed with the PROCHECK software. (Figure 1) depicts the evaluation of the SWISS-MODEL generated model assessment. The graph shows that the yellow regions are the most permissible. Other residues are expressed by squares, while glycine is represented by triangles. The modeled structure has 87.3% residue in the most favored region, according to satisfactory results. Molecular docking (Structure-Based Virtual Screening) is a popular method for repurposing the drug here we focus on the effects of natural compounds from the HIT compounds library against spike E2 protein. We have used this method to find the top 10 compounds from the ZINC database subset HIT library, which has a total of 707 compounds and screened selected compounds based on the lowest binding free energies (Table 1). These top hit compounds screened as a potential inhibitor against spike E2 protein which binds to the active pocket of this protein.

Lipinski's rule of five properties and ADMET profile predicted by software found that Guttic acid had passed successfully and they were found to be safe (Table 2). Further, interaction analyses of the docked natural compounds explored the functional interactions with the active site of the spike E2 protein. The docking poses of these compounds (Figure 2) were stabilized by the same hydrophobic interactions involving 4 amino acid residues GLY44, ARG104, CYS105, HIS131. Gambogic acid was found to be the persuasive inhibitor of spike E2 protein; the biological properties are antitumor, antioxidant, and anti-inflammatory properties. If we stay away from the excessive doses that induce toxicity, it does not have any adverse side effects. Gambogic acid is a natural phenolic compound that has a xanthone backbone. The main source is Garcinia hanburyi, an evergreen tree from Clusiaceae family.

## Conclusion:

The simulation study shows that Gambogic acid could be a potential candidate against the Chikungunya virus. The mechanism of action includes the interaction-binding of Gambogic acid with the E2 protein of the Chikungunya virus. Cell and animal model-based studies could validate the findings of the present study along with setting safe doses of the Gambogic acid for humans.

## Funding:

No specific funding was available for this research

## Figures and Tables

**Table 1:** Docking Score selected top 10 hit obtained from Virtual screening of Virus E2 protein with (Herbal ingredient target) database.

**S.No.**	**Name of the ligand**	**Binding Free Energy (kcal/mol)**	**pKi**	**Ligand Efficiency (kcal/mol/ non-H atom)**	**Torsional**
1	ZINC000100080550	-10.9	7.99	0.1879	3.113
2	ZINC000100230355	-10.8	7.92	0.2	2.4904
3	ZINC000100230359	-10.8	7.92	0.2	2.4904
4	ZINC000100230363	-10.8	7.92	0.2	2.4904
5	ZINC000100230367	-10.8	7.92	0.2	2.4904
6	ZINC000095098823	-10.3	7.55	0.2711	0.6226
7	ZINC000118913578	-10.2	7.48	0.3091	0.3113
8	ZINC000118913574	-10.1	7.41	0.2971	0.6226
9	ZINC000150338757	-10	7.33	0.1205	5.6034
10	ZINC000030726863	-9.9	7.26	0.202	0.6226

**Table 2:** ADMET properties of selected compounds Guttic acid

**ADMET**	**Predicted properties**	**Value**	**Probability**
A (Absorption)	Human intestinal absorption (HIA)	+	0.921
	Human oral bioavailability (HOB)	-	0.6571
	Caco-2 permeability	+	0.8041
D (Distribution)	Plasma protein binding (PPB)	1.337	100%
	P-glycoprotein substrate, an inhibitor	+	0.8501
	Blood-brain barrier penetration (BBB)	+	0.9514
M (Metabolism)	Substrate: CYP2C9, 2D6	-	0.8689
	3A4	+	0.7023
	Inhibitor:2D6, 2C9, 2C19, 3A4	-	0.8818
	CYP1A2	+	0.5364
E (Excretion)	Half-time (t1/2)		
	Renal clearance		
T (Toxicity)	Carcinogenicity (binary)	-	0.9714
	(hERG) inhibition	-	0.449
	Acute toxicity	I	0.4789
	Eye injury & Eye corrosion	-	0.9916
	Biodegradation	-	0.9
